# Immunotherapy in Metastatic Renal Cell Carcinoma: A Comprehensive Review

**DOI:** 10.1155/2015/367354

**Published:** 2015-06-16

**Authors:** Rachna Raman, Daniel Vaena

**Affiliations:** Division of Hematology Oncology and Bone Marrow Transplantation, Department of Internal Medicine, University of Iowa Hospitals and Clinics, 200 Hawkins Drive C32GH, Iowa City, Iowa 52242, USA

## Abstract

Localized renal cell carcinoma (RCC) is often curable by surgery alone. However, metastatic RCC is generally incurable. In the 1990s, immunotherapy in the form of cytokines was the mainstay of treatment for metastatic RCC. However, responses were seen in only a minority of highly selected patients with substantial treatment-related toxicities. The advent of targeted agents such as vascular endothelial growth factor tyrosine kinase inhibitors VEGF-TKIs and mammalian target of rapamycin (mTOR) inhibitors led to a change in this paradigm due to improved response rates and progression-free survival, a better safety profile, and the convenience of oral administration. However, most patients ultimately progress with about 12% being alive at 5 years. In contrast, durable responses lasting 10 years or more are noted in a minority of those treated with cytokines. More recently, an improved overall survival with newer forms of immunotherapy in other malignancies (such as melanoma and prostate cancer) has led to a resurgence of interest in immune therapies in metastatic RCC. In this review we discuss the rationale for immunotherapy and recent developments in immunotherapeutic strategies for treating metastatic RCC.

## 1. Introduction

Renal cell cancer (RCC) is the sixth most common malignancy in men and the eighth most common malignancy in women in the United States. The incidence of RCC rose by 1.6% per year between 2002 and 2011 with 63,920 new cases and 13,860 deaths anticipated in 2014 [1]. More than a decade ago, immunotherapy with cytokines was the standard treatment for metastatic RCC (mRCC). Subsequently, targeted agents such as vascular endothelial growth factor tyrosine kinase inhibitors (VEGF-TKIs) and inhibitors of mammalian target of rapamycin (mTOR) showed significantly improved responses and progression-free survival (PFS). These agents were also relatively well tolerated, thereby changing the treatment paradigm for metastatic RCC. Comparison of disease specific survival for de novo metastatic RCC between 1992–2004 (pretargeted therapy) and 2005–2009 (era of targeted therapies) showed an improvement from 13 months to 16 months (*P* < 0.0001) [2]. Upon further risk stratification, sequential use of VEGF-TKIs may yield median survivals of 43, 22, and 7.3 months in favorable-, intermediate-, and poor-risk groups, respectively [3]. Yet only 12% of patients with metastatic RCC are alive at five years, with the majority eventually developing treatment resistance and disease progression. In contrast, cytokine therapy with high-dose interleukin 2 may achieve a complete response in 7–10% of cases with some persisting beyond 10 years [4], thereby “curing” a subset of patients of their disease. However, no significant improvement in overall survival occurs and severe toxicities limit their clinical utility. In the last few years, new immunotherapeutic targets have been identified with reports of durable responses, improved overall survival, and better tolerability. In this review we discuss the rationale for immunotherapy, current status of cytokine therapy, status of biomarkers to improve patient selection, and recent advances in immunotherapy for metastatic RCC. For the purpose of this review, mRCC refers to clear cell histology only.

## 2. Rationale for Immunotherapy in Renal Cell Cancer

Reports of spontaneous regressions, prolonged disease stability, and late relapses after nephrectomy suggest an inherent role of immune mechanisms in the natural history of RCC [5–8]. In keeping with these anecdotal reports, diffuse tumor infiltration with T cells, natural killer (NK) cells, dendritic cells (DCs), and macrophages have been described in RCC [9–11], but the precise role of each cell type is not well understood. Yet, most tumors are not eliminated by immune effector cells, possibly because of the incompletely understood mechanisms of immune tolerance. Most antigens expressed by tumor cells are merely overexpressed normal self-antigens. Moreover, tumor cells act as poor antigen-presenting cells. Thus, the repertoire of cytotoxic T cells (CTLs) in the host that recognize the tumor antigens as foreign is probably small. Mapara and Sykes [12] comprehensively reviewed the basic principles of immune tolerance to tumors as summarized in the context of RCC in Table 1.

The ensuing sections discuss the past and current developments to overcome tumor mediated immune evasion in metastatic RCC. Broadly, these include (1) T cell modulation, for example, with cytokines and immune checkpoint inhibitors, (2) adoptive cellular immunotherapy, and (3) vaccination.

## 3. Immunotherapy for Renal Cell Cancer: Past and Current Developments

### 3.1. T Cell Modulation

#### 3.1.1. The Current Status of Cytokine Therapy in Metastatic RCC

The two principal cytokines with proven efficacy in metastatic renal cancer are interferon-alpha (INF-*α*) and high-dose interleukin 2 (IL2). IL2 is a potent stimulator of T cell proliferation and differentiation, while INF-*α* has antiangiogenic effects, promoting antigen presentation and dendritic cell maturation [13]. However, their exact mechanism of action is unknown.

High-dose IL2 was approved for mRCC in 1992. Long-term follow-up of 255 patients with mRCC enrolled in seven phase II clinical trials of high-dose IL2 reported objective responses in 15% including complete responses (CR) in 7% of patients. IL2 was administered at 600,000 IU/kg for 14 doses or at 720,000 IU/kg for 12 doses every 8 hours per treatment week. For the complete responders the median duration of response was at least 80 months (range 7–>131 months). Median survival time for all 255 patients remained 16.3 months as of 2000 [4]. In addition to reversible toxicities, a 3-4% treatment related mortality was a deterrent to widespread use. Efforts to minimize toxicity while improving response rates with IL2 have included dose reductions, schedule changes, combination of interferon and sorafenib, and chemotherapy which either did not improve response rates significantly or improved responses at the cost of increased toxicity [14–18]. The combination of sorafenib and bevacizumab with cytokines has been used with some success in renal cell cancer. However, these combinations do not appear to produce more durable responses than cytokines alone [19–24]. Clinical benefit and durable CRs following high-dose IL2 administration were also recently reported after prior use of TKIs [25].

Several retrospective studies have evaluated predictors of efficacy or resistance to cytokines and proposed various clinical, serological, and histologic biomarkers. Risk models developed in accordance with these biomarkers are listed in Table 2. In addition to these models, a correlation between response to cytokines and serum levels of VEGF and fibronectin has also been suggested [26].

The cytokine working group undertook the “SELECT” trial [30, 31] to prospectively evaluate whether the available risk stratification tools and biomarkers were predictive of response to high-dose IL2. Of the models shown in Table 2, ISM or MSKCC scores were unable to improve selection criteria. No responses were seen in the high UCLA SANI risk group and non-clear-cell RCC. Interestingly, response (including durable response lasting more than 3 years) was positively associated with tumor expression of PD-L1 or B7-H1 (programmed death ligand) by IHC staining [32].

Despite clinical benefit in a minority of patients the durability of responses seen with high-dose IL2 is yet to be surpassed by currently available VEGF-TKIs in mRCC [33]. However toxicity remains a concern and there is a lack of robust tools to predict benefit in an individual patient. Efforts to maximize clinical benefit with better tolerated therapy have led to renewed interest in developing “targeted immunotherapy.”

#### 3.1.2. Immune Checkpoint Inhibitors

**Figure 1:**
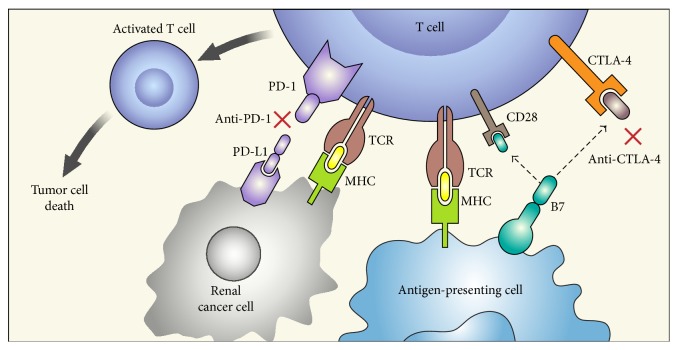
Mechanism of action of immune checkpoint inhibitors. PD-1 is expressed on activated T cells and when it binds to its ligand PD-L1 on tumor cells leads to T cell exhaustion. CTLA-4 competes with CD28 (costimulatory T cell molecule) for B7 ligands (CD80 and CD86 that are not shown in the figure) and upon activation decreases T cell proliferation as well as activity. Blockade of CTLA-4 (by anti-CTLA-4) and PD-1 (anti-PD-1) or PD-L1 stimulates effector T cells to produce antitumor responses. Adapted by permission from Macmillan Publishers Ltd. [37], copyright (Jan 2014). PD-1: programmed death-1, PD-L1: programmed death-ligand 1, MHC: major histocompatibility complex, TCR: T cell receptor, and CTLA-4: cytotoxic T lymphocyte antigen.

Checkpoint receptors (CPRs) on cytotoxic T lymphocytes (CTLs) block costimulatory signals at various stages of immune activation after ligand binding. This results in T cell anergy and immunosuppression. Blocking these CPRs appears to improve the ability of CTLs to mount and sustain an effective T cell response. Cytotoxic T lymphocyte antigen (CTLA-4) is a CPR on T cells that ligates to B7 molecules (CD80 and CD86) on antigen-presenting cells (APCs) and inhibits T cell proliferation as well as function. Programmed death-1 (PD-1) is another T cell receptor which is expressed on activated, antigen-exhausted T cells and binds to its ligands, PD-L1 (B7-H1) and PD-L2 (B7-DC), thereby inducing anergy. While PD-1 is expressed primarily on mononuclear cell infiltrates, PD-L (PD-L1 being the predominant ligand) is expressed by tumor cells. Among the solid tumors, PD-L1 expression has largely been demonstrated in melanoma, non-small-cell lung cancer, and RCC cells and correlates with poor outcomes when treated with existing systemic therapies [34, 35]. Blocking the PD-1 pathway enhances immune responses by stimulating effector T cells in the tumor and its microenvironment. Alternatively, it may also decrease the number or suppressive activity of regulatory T cells [36] (Figure 1).

*(A) Cytotoxic T Lymphocyte Antigen-4 (CTLA-4) Antibody.* Ipilimumab, a monoclonal antibody directed against CTLA-4, was the first drug that was shown to produce a survival benefit in advanced melanoma [38]. In a single institution phase II study of ipilimumab in metastatic RCC, 5 of 40 responses were noted in the higher dose group (3 mg/kg every 3 weeks) compared to 1 of 21 responses in the lower dose group (3 mg/kg followed by 1 mg/kg every 3 weeks). Interestingly a significant association was observed between autoimmune events and tumor regression (30% with AE versus 0% without AE). Though all responses were partial, patients who had failed IL2 also responded [39]. To our knowledge, there are currently no other studies of single agent ipilimumab in RCC and the ongoing trials are evaluating the efficacy of combining ipilimumab with PD-1 blockade in RCC [40]. A phase III study of ipilimumab and the anti-PD-1 antibody nivolumab versus sunitinib is currently recruiting patients with previously untreated advanced or metastatic RCC (CheckMate 214).

Although a phase I study of another CTLA-4-directed monoclonal antibody, tremelimumab in combination with sunitinib, showed RR of 43% in metastatic RCC the combination was not recommended for further investigation due to rapid onset renal failure noted in a subset of patients [41].

*(B) Programmed Death-1 Inhibitors (PD-1).* Nivolumab (previously BMS 936558 and MDX-1106) is a fully humanized PD-1 blocking antibody. Promising responses, some durable, have been reported in phase I and II studies in melanoma, non-small-cell lung cancer [42, 43], and, more recently, renal cell cancer.

A phase I study of nivolumab in patients with relapsed or refractory solid tumors demonstrated its safety and clinical efficacy as a single infusion of 0.3, 1, 3, or 10 mg/kg [42]. Subsequently, nivolumab was tested in a larger study with 296 patients that included 34 patients with metastatic renal cell carcinoma (most had received two or more prior regimens). Objective responses occurred in 4 of 17 patients (24%) treated with a dose of 1.0 mg/kg and in 5 of 16 (31%) treated with 10.0 mg per kilogram. Of the responding patients, more than 50% had responses lasting a year or more with one being a complete response (6%). Nine patients (27%) had stable disease beyond 24 weeks. Infusion reactions which were mostly grades I and II were managed by glucocorticoids and antihistamines. Most of the other adverse events which included rash, hypothyroidism, hepatitis, nausea, adrenal insufficiency, diarrhea, and vitiligo were grades I-II. Hypopituitarism was observed in less than 1%. Grades III-IV immune mediated toxicities specifically pneumonitis were observed in 14/296 patients [44]. Results of the phase II study assessing the efficacy of nivolumab at three dose levels (0.3, 2, or 10 mg/kg IV every 3 weeks) in 168 patients with previously treated mRCC were recently presented at ASCO 2014 [45]. No dose-response relationship for PFS was observed and a response rate of at least 20% was observed at all doses. Despite an unimpressive PFS, responses were durable and persisted for about 2 years. Improved OS was noted with doses of 2 and 10 mg/kg (25.5 and 24.7 months, resp., versus 18.2 months for 0.3 mg/kg).

With the demonstration of safety and antitumor activity of PD-1 blockade, ongoing trials are evaluating combinations of agents with activity in RCC. VEGF-TKIs may augment the antitumor efficacy of PD-1 blockade by reducing the percentage of tumor infiltrating regulatory T cells and enhancing the activity of CTLs [46–48]. Combinations of nivolumab with sunitinib or pazopanib [49], bevacizumab (NCT02210117), and the anti-CTLA-4 antibody ipilimumab [40] are currently undergoing clinical testing. Results of the phase I study evaluating the combination of nivolumab with sunitinib or pazopanib in previously treated mRCC were presented at ASCO 2014 [49]. Overall response rate was 52% with sunitinib and 45% with pazopanib. Dose limiting liver toxicity was noted in the pazopanib arm leading to its closure. PFS at 24 weeks was 78%, which however is comparable to sunitinib alone in the first-line treatment of metastatic RCC [50]. In another study, the combination of nivolumab and ipilimumab showed a response rate of 45% [40] with an acceptable safety profile. Durability of responses with these combinations should be assessed in phase III studies.

Other PD-1 and PD-L1 inhibitors such as pembrolizumab (MK-3475) and pidilizumab (CT-011) are also under evaluation (Table 3).

As results of efficacy, tolerability, and durability of responses with immune checkpoint inhibitors (specifically with PD-1) emerge, efforts to guide patient selection are also underway. In this context, PD-L1 expression has been proposed as a potential biomarker of response. It was recently shown that responses across multiple cancer types (including RCC) were observed in tumors expressing high levels of PD-L1, especially when PD-L1 was expressed by tumor-infiltrating immune cells [51]. In the phase II study of single agent nivolumab for previously treated RCC, 31% responses were seen in PD-L1 positive tumors compared to 18% in PD-L1 negative RCC [45]. However, in the combination studies of nivolumab with ipilimumab, sunitinib, or pazopanib, a significant proportion of patients with PD-L1 negative tumors also responded to the treatments. Thus, the precise role of PD-1/PD-L1 expression as a biomarker is yet to be defined.

### 3.2. Adoptive Cellular Immunotherapy

Adoptive cellular immunotherapy (ACI) entails in vitro expansion of immune effectors (autologous or allogeneic lymphocytes) with antitumor activity and reinfusing them into the tumor bearing host. First described in RCC in 1992, ACI has thereafter been evaluated in several clinical studies with or without cytokines [52–55]. Conflicting data on efficacy, significant cost, and a labor intensive process of preparation has limited the pace of development of ACI in RCC.

### 3.3. Vaccine Therapy

Vaccines carry tumor antigens on a vehicle that may be a cell, peptide, or a vector. They are designed to enhance innate or adaptive immunity depending on the antigen and vehicle. Examples include autologous tumor cell vaccines, dendritic cell (DC) based vaccines, and peptide based vaccines. Results from ongoing trials of DC vaccines in RCC are the most promising and are discussed here.

The efficacy and success of sipuleucel T in metastatic prostate cancer [56] prompted the evaluation of dendritic cell (DC) vaccines in metastatic renal cell cancer. DCs play a critical role in producing antitumor immunity. Although mature DCs are potent stimulators of CTLs and natural killer cells (NKCs), immature DCs may tolerize the T cells and decrease their antitumor responses [12]. In vivo, DCs are often inefficient APCs; hence peptide vaccines that rely on DCs may not induce a strong enough antitumor immune response. To constitute these vaccines DCs are allowed to undergo maturation ex vivo in the presence of tumor antigens and then infuse into the tumor bearing host. Phase I studies of vaccines containing DCs transfected with tumor RNA or pulsed with tumor lysate found them to be safe and effective in RCC either alone [57–59] or in combination with cytokines [60, 61].

The most compelling evidence for the efficacy of dendritic cell vaccines came from the phase II study of AGS-003 with sunitinib in de novo metastatic RCC. Updated results were presented in the 2014 Annual Meeting of American Society of Clinical Oncology [50, 62]. The production of AGS-003 is a multistep process and starts with leukapheresis to collect DCs from the tumor bearing host. AGS-003 is manufactured by transfecting the autologous DCs with patient-specific RCC tissue amplified RNA and synthetic-truncated human CD40 ligand RNA, which has the potential to stimulate the immune system. The vaccine is then reintroduced into the patient intradermally, eliciting a highly specific CTL response through the initiation of a signaling cascade that causes the secretion of the cytokine IL-12. In this study, 21 patients with newly diagnosed unfavorable-risk (time from diagnosis to treatment of less than 1 year) mRCC received sunitinib plus AGS-003. The median PFS was 11.2 months and the median OS was 30.2 months. 52% patients survived beyond 30 months, 23% of them still alive after 5 years. When responses were analyzed by baseline Heng risk status [3], patients in the intermediate-risk group (*n* = 11) had an OS of 57 months and poor-risk patients (*n* = 10) had OS of 9.1 months (ranged up to 56.3 months). The absolute change in CD8+CD28+ memory T cells directly and significantly correlated with prolonged OS and PFS. This was a marked improvement from a median OS of 22.5 months for intermediate-risk patients and 7.8 months for poor-risk patients for patients treated with VEGF-TKIs [63]. No additive toxicity other than grades I and II infusion site reactions was noted.

The rationale for combining vaccine therapy with sunitinib comes from the observed favorable effects of VEGF-TKIs on reversing immunosuppression by decreasing Tregs and myeloid derived suppressor cells in the tumor microenvironment [64]. The promising results from the phase II trial have prompted an ongoing phase III study of this combination (NCT01582672/ADAPT). The ADAPT clinical study is a randomized trial, where the experimental arm would receive a combination of AGS-003 and a first-line targeted therapy, starting with sunitinib. The comparator arm would receive standard treatment beginning with sunitinib alone. After 6 weeks of targeted therapy beginning with sunitinib, patients will receive 8 doses of AGS-003 during the first year and for those continuing to benefit after the first year of treatment, booster doses of AGS-003 will be given every 3 months thereafter, in combination with standard targeted therapy. The primary end point of the trial is overall survival.

Despite the constraints of cost and the multistep constitution process, their relative safety and early results showing unprecedented outcomes in mRCC warrant continued evaluation of therapeutic vaccines in phase III studies.

## 4. Conclusion

Renewed interest in reprogramming the immune system to improve the outlook for metastatic RCC has led to evaluation of several immune checkpoint inhibitors and vaccination strategies in multiple ongoing trials. The OS reported with the PD-1 inhibitor nivolumab in previously treated mRCC has already exceeded the median OS reported with IL2 in the first-line treatment of mRCC. Manageable toxicities and wider applicability add to its appeal. Clinical benefit over and above that with TKIs is yet to be proven as is the durability of responses comparable to IL2. Combinations of PD 1 inhibition with VEGF-TKIs and CTLA-4 inhibitors have shown significantly higher response rates, though the safety of these combinations is in question. Meaningful clinical benefit has been observed with these checkpoint inhibitors in heavily pretreated patients with mRCC which has significant implications. The 5-year follow-up results from the phase II study of AGS-003 in combination with sunitinib have shown an unprecedented survival in mRCC regardless of the risk category. Results of the phase III study are eagerly awaited. In our opinion, addition of these newer modulators of immunity to the available treatments for management of mRCC (VEGF-TKIs, mTOR inhibitors, and surgical cytoreduction) may significantly alter the long-term outcomes in mRCC. It would be of significant clinical interest to simultaneously evaluate appropriate treatment sequencing and tools to improve on patient selection. Until long-term data on the durability of treatment responses are available, IL2 may still be considered in a small group of otherwise healthy patients with mRCC who have a low disease burden. However, when appropriate, participation in clinical trials evaluating immune modulation must be encouraged.

## Figures and Tables

**Table 1:** Proposed mechanisms of tumor mediated immune evasion.

Effects of tumor mediated immune evasion on T cells	Molecular mechanisms underlying tumor effects on T cells
Direct deletion of immune effector cells	Expression of death inducing ligand (Fas)
	Secretion of immunosuppressive cytokines: IL-10, TGF*β*

Direct tolerization of tumor reactive T cells	Cross presentation of tumor antigens by bone marrow APCs
	B7-H1 expression by tumor and induction of T cell apoptosis

Inhibition of T cell activation or induction of anergy	Lack of expression of costimulatory molecules (CD 28 on T cells, B7 ligands on APCs)
	Overexpression of inhibitory costimulatory molecules CTLA-4, PD-1, and PD-1 ligands

IL: interleukin, TGF: tissue growth factor, APCs: antigen-presenting cells, B7-H1: B7-homolog 1, CTLA-4: cytotoxic T lymphocyte antigen, PD1: programmed death-1, PD-L1: programmed death-ligand 1, and VEGF: vascular endothelial growth factor.

**Table 2:** Risk stratification models in cytokine treated metastatic RCC.

Risk models	Model factors	Outcomes
MSKCC [27]	KPS <80% LDH 1.5x ULN Hemoglobin < LLN Corrected calcium > ULN Interval from diagnosis to treatment of <1 year	**Median OS (months)** Favorable: 30 Intermediate: 14 Poor: 5

UCLA SANI [28]	Lymph Node status Constitutional symptoms Location of metastasis Sarcomatoid histology TSH	**5-year OS (%) and ORR (%)** Low risk: 41 and 43 Intermediate: 19 and 27 High: 0 and 15

ISM [29]	Histology: clear cell with alveolar features absence of papillary or granular features CA-9 expression by IHC	Good risk accounted for 96% of responding patients and 56% of nonresponding patients

KPS: Karnofsky performance status, ULN: upper limit of normal, LLL: lower limit of normal, ISM: integrated selection model, ORR: overall response rate, CA-9: carbonic anhydrase-9, and IHC: immunohistochemistry.

**Table 3:** Programmed death (PD-1 and PD-L1) inhibitors in various phases of development.

Agent	Description	Target	Phase of development	Being tested in RCC	Trial identifier
BMS 936558/MDX-1106/nivolumab	Human IgG monoclonal Ab	PD-1	I, II, and III	Yes	NCT01472081 NCT01354431 NCT01668784 NCT02210117 NCT02231749

MK-3475/pembrolizumab	Human IgG4 monoclonal Ab	PD-1	I and II	Yes	NCT01704287 NCT02318771 NCT02212730 NCT02133742 NCT01295827 NCT02089685 NCT02014636

CT-011^*^/pidilizumab	Human IgG1 monoclonal Ab	PD-1	II	Yes	NCT01441765

MPDL3280A	Monoclonal Ab	PD-L1	I and II	Yes	NCT01375842 ^**^ NCT01633970

BMS-936559/MDX1105-01	Human IgG4 monoclonal Ab	PD-L1	I	Yes	NCT00729664

AMP-224	B7-DC/IgG1 fusion protein	PD-1	I	Yes	NCT01352884

Ab: antibody, DC: dendritic cell, PD: programmed death, and RCC: renal cell cancer. ^*^PD-1 blockade alone or in combination with the dendritic cell (DC)/renal cell carcinoma (RCC) fusion cell vaccination. ^**^ Phase II comparing MPDL3280A monotherapy or in combination with bevacizumab versus sunitinib in patients with previously untreated locally advanced or metastatic RCC.
